# Short-term outcomes of modified Y-graft technique in acute type a aortic dissection using the femoral artery bypass and one minute systemic circulatory arrest technique

**DOI:** 10.1186/s13019-020-01156-5

**Published:** 2020-05-20

**Authors:** Xiangfei Sun, Qi Zhao, Yufeng Huo, Jinfeng Zhou, Fen Zhao, Yimin Liu, Yonghai Du, Songxiong He, Chao Liu, Detian Jiang, Wenyu Sun

**Affiliations:** 1 Department of Cardiovascular Surgery, Shandong Provincial Hospital, Cheeloo College of Medicine, Shandong University, Jinan, Shandong 250021 China; 2 Department of Cardiovascular Surgery, Shandong Provincial Hospital Affiliated to Shandong First Medical University, Jinan, Shandong 250021 China; 3 Department of Gastroenterology, Shandong Provincial Hospital, Cheeloo College of Medicine, Shandong University, Jinan, Shandong 250021 China; 4 Department of Gastroenterology, Shandong Provincial Hospital Affiliated to Shandong First Medical University, Jinan, Shandong 250021 China; 5 Department of Cardiovascular Surgery, Qilu Hospital of Shandong University, Qingdao, 266011 Shandong People’s Republic of China

**Keywords:** Modified Y-graft technique, Femoral artery bypass, One minute systemic circulatory arrest, Acute type a aortic dissection

## Abstract

**Objective:**

Aortic arch replacement in acute type A aortic dissection patients remains the most challenging cardiovascular operation. Herein, we described our modified Y-graft technique using the Femoral Artery Bypass (FAB) and the One Minute Systemic Circulatory Arrest (OSCA) technique, and assessed the short-term outcomes of the patients.

**Methods:**

Between February 2015 and November 2017, 51 patients with acute type A aortic dissection underwent aortic arch replacement. Among them, 23 patients underwent FAB while 28 patients underwent both FAB and OSCA. The intraoperative data and postoperative follow-up data were recorded. The follow-up data of patients with traditional Y-graft technique were collected from previously reported studies.

**Results:**

In the FAB group, two patients died due to pulmonary infection (30-day survival rate, 91.3%), and two patients were paralyzed from the waist down. Hemodialysis was performed for five patients (21.7%) before hospital discharge. Fifteen patients (65.2%) received respiratory support for more than 2-days and eight patients (34.8%) for more than 5-days. These follow-up results were comparable or better than the patients with traditional Y-graft technique. Furthermore, compared to the FAB group, the morbidity due to neurological dysfunction and acute renal failure was significantly reduced in the FAB+OSCA group. Moreover, the respiratory support, length of postoperative stay and ICU stay were shortened.

**Conclusions:**

This study clarified the feasibility of FAB and OSCA technique in modifying Y-graft technique. The acute type A aortic dissection patients showed less surgical complications and favorable short-term outcomes after this surgery.

## Introduction

The first successful aortic arch replacement was reported more than fifty years ago. With the development of the surgical technique and the improvement of patient care, the aortic arch can be repaired more safely now. However, total aortic arch replacement in acute type A aortic dissection patients remains the most challenging cardiovascular operation, which incurs high risk of cerebral damage and acute renal failure, and consequently, a considerable risk of operative mortality. The 30-day mortality was as high as 18% and stroke rates were as high as 10% in 2011 [1].

In 2002, Spielvogel and colleagues developed the Y-graft technique to enable antegrade selective cerebral perfusion [2]. The individual reconstruction of brachiocephalic branches using a trifurcated graft benefitted the prognosis of the patients [3, 4]. However, the outcome of the aortic arch replacement surgery is largely affected by the surgeon’s skills and the tentative surgery techniques [5, 6]. Hence, feasible and beneficial modifications of this technique are urgently needed.

Our team modified the traditional Y-graft technique using Femoral Artery Bypass (FAB) and One Minute Systemic Circulatory Arrest (OSCA) technique to simplify arch reconstruction, reduce embolization and avoid cerebral ischemia.

In the past 2 years, we have treated 51 cases of acute type A aortic dissection with our modified Y-graft technique for aortic arch replacement. The perioperative data and short-term follow-up data, such as perfusion time, 30-days survival rate, neurological dysfunction, length of respiratory support and acute renal failure, were recorded for all patients. The follow-up data of patients with traditional Y-graft technique were collected from previously reported studies. Herein, we described our modified Y-graft technique and assessed the short-term outcomes of the patients.

## Patients and methods

### Patients

We conducted a retrospective analysis of the data from 51 patients who underwent aortic arch replacement between February 2015 and November 2017 at our hospital. The clinical features of the patients are shown in Table 1. Of these patients, 23 (45.1%) underwent modified Y-graft technique using the FAB technique, and 28 (54.9%) underwent modified Y-graft technique using the FAB+OSCA techniques. The perioperative data and short-term follow-up data were recorded for all patients. Written informed consent was obtained from each patient. This study was approved by the Ethics Committee of Qilu Hospital of Shandong University.

**Table 1:** Patient characteristics

Characteristic	Value
Number of patients	51
Age, years	46.3 ± 9.9
Sex, men: women	42:9
Emergency operation	49 (96.1%)
Marfan syndrome	4 (7.8%)
Aortic valve regurgitation	18 (35.3%)
Diabetes mellitus	1 (2.0%)
Smoking, past or current	41 (80.4%)
Hypertension	48 (94.1%)
Renal dysfunction	3 (5.9%)
Pulmonary disease	1 (2.0%)
Ischemic coronary heart disease	4

Data are reported as mean ± SD, median (interquartile range), or number (%)

### Surgical technique

The surgical procedure was divided into three stages.

#### Stage 1: reconstruction of three branches (using FAB technique, Fig. 1)

The femoral artery was exposed through a small incision in the inferior inguinal ligament. The left axillary artery was exposed through a small infra-clavicular incision. A median sternotomy was performed with extension of the incision superiorly along the medial border of the left sternocleidomastoid muscle and the brachiocephalic vessels were exposed.

**Fig. 1:**
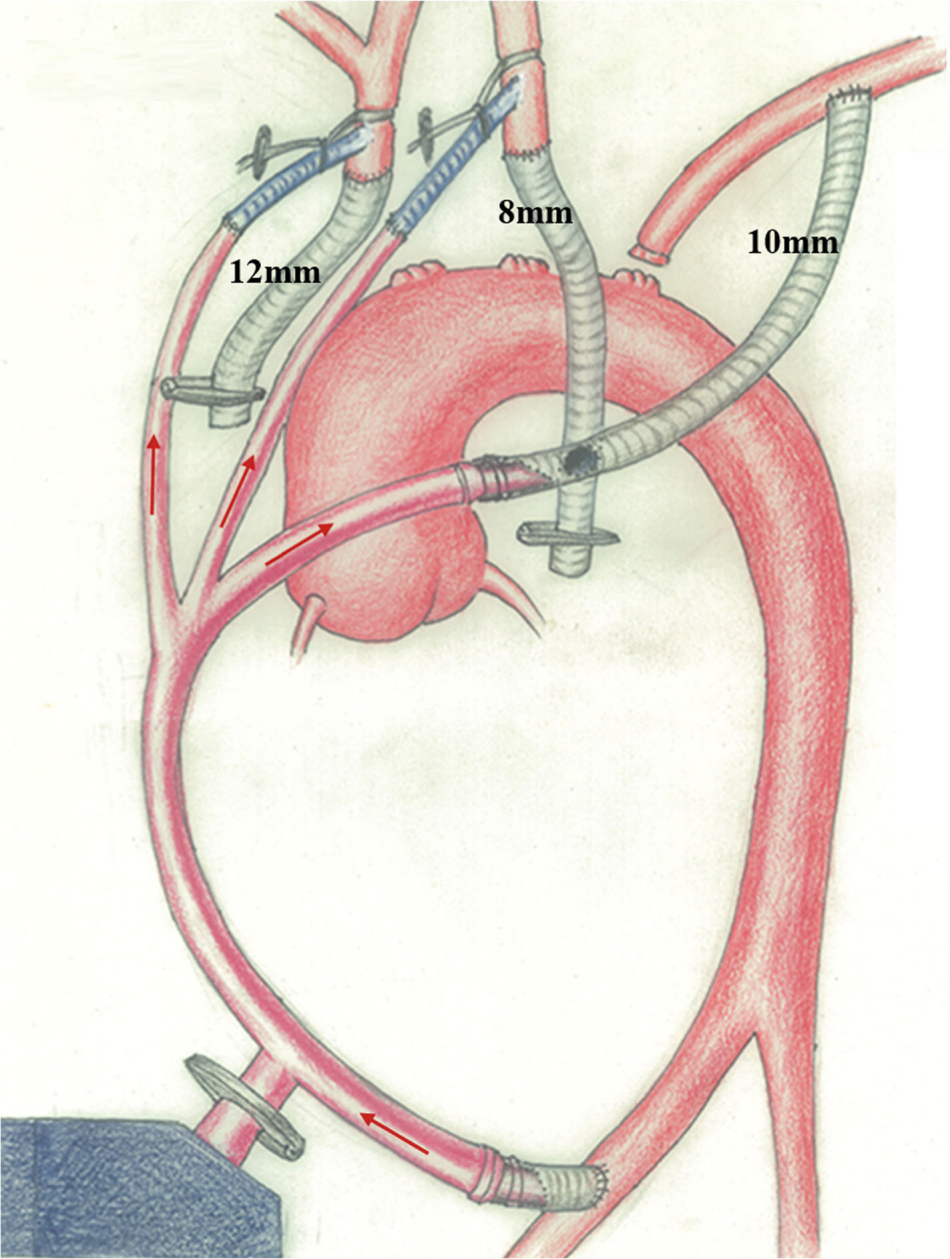
Reconstruction of three branches using femoral artery bypass (FAB) technique. The construction of three bypasses: femoral artery-to-left common carotid artery bypass, femoral artery-to-left subclavian artery bypass and femoral artery-to-innominate artery bypass

Intravenous heparin was administered to achieve an activated clotting time (ACT) > 350 s. A 10 mm graft (4 cm length) was end-to-side anastomosed to the femoral artery and then connected to the arterial tubes of the cardiopulmonary bypass (CPB) machine. Another 10 mm (15 cm length) graft was end-to-side anastomosed to the left subclavian artery and the other end of this graft was tunneled via the second intercostal space into the mediastinum on demand. The left common carotid artery was cannulated with an arterial catheter, which was connected to the arterial tubes of the CPB machine. Since the CPB machine did not work, the femoral artery-to-left common carotid artery bypass was completed. Then the left common carotid artery was transected. Another 8 mm graft was end-to-end anastomosed to the left common carotid artery. The previous 10 mm graft (connected to the left subclavian artery) was measured to the appropriate length and side-to-side anastomosed to this 8 mm graft (connected to the left common carotid artery). The free-end of the previous 10 mm graft (connected to the left subclavian artery) was tightly connected to the arterial tubes of the CPB machine. The free-end of the 8 mm graft (connected to the left common carotid artery) was clamped. This is how both femoral artery-to-left common carotid artery bypass and the femoral artery-to-left subclavian artery bypass were completed. As for the innominate artery, the procedures of cannulation and anastomosis were similar to the left common carotid artery. The innominate artery was cannulated with an arterial catheter, which was connected to the arterial tubes of the CPB machine. A femoral artery-to-innominate artery bypass was completed. Then the innominate artery was transected. A 12 mm graft was end-to-end anastomosed to the innominate artery. The free-end of the 12 mm graft was clamped.

Finally, without the assistance of CPB machine, femoral artery-to-left common carotid artery bypass, femoral artery-to-left subclavian artery bypass and femoral artery-to-innominate artery bypass were all completed at room temperature, with continuous selective bilateral cerebral perfusion. The reconstruction of the three branches was also successfully completed. Every graft was carefully de-aired, and perfusion was restored to the head and upper extremities (Fig. 1).

#### Stage 2: reconstruction of the proximal aortic root

The pericardium was opened after cannulation of the superior and inferior vena cava, and ensuring that the CPB machine worked and cooling was started. The ascending aorta was cross-clamped at 32 °C and the cardioplegic solution was usually perfused through a coronary sinus cannulation to arrest the heart. After the cardioplegic cardiac arrest, the aortic valve repair or replacement (Bentall) was performed if significant aortic valve insufficiency was identified through TEE. The ascending aorta was also replaced by the graft, which was anastomosed to the aortic sinotubular junction or the artificial valve ring.

#### Stage 3: reconstruction of the aortic arch (using OSCA technique, Fig. 2)

**Fig. 2:**
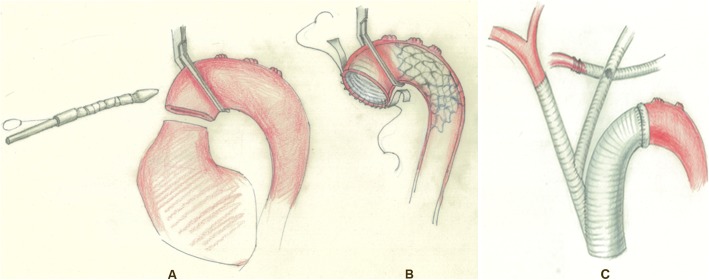
One minute systemic circulatory arrest (OSCA) during the surgery. **a** The intraoperative stent was placed into the distal aortic arch. **b** The aortic arch was immediately cross-clamped after de-airing, which greatly shortened the duration of the hypothermic circulatory arrest to about 1 min. **c** The 8 mm graft was end-to-side anastomosed to the 12 mm graft (innominate artery). The free-end of the 12 mm graft was end-to-side anastomosed to the ascending graft in ideal site

The completion of the reconstruction of the proximal aortic root usually coincided with the end of core cooling (target temperature: 32 °C). At the beginning of this stage, the patient was placed in slight Trendelenburg position, and the head was packed in ice. The cross-clamp was moved to the distal aortic arch (between the innominate artery and the left common carotid artery), and the aortic arch was trimmed. Hypothermic circulatory arrest was started after removing the clamp. The intraoperative stent (Stented Graft System In Surgical Operation) was placed into the distal aortic arch and then the aortic arch was immediately cross-clamped after de-airing (Fig. 2a). Thereafter, the lower-body perfusion was restored, and active rewarming was started. The duration of the hypothermic circulatory arrest was about 1 min. The subsequent steps could be performed in an unhurried fashion. The proximal self-aortic arch and the intraoperative stent’s graft section were trimmed, and the sandwich treatment was performed (Fig. 2b). The previously placed graft on the root was retracted, stretched and measured to the site of distal anastomosis. Graft-to-graft anastomoses were performed. Then the aortic cross-clamp was removed.

Before the cannulation of the innominate artery and removal of the graft connected to the left subclavian artery, the 8 mm graft (left common carotid artery) was end-to-side anastomosed to the 12 mm graft (innominate artery). The free-end of the 12 mm graft (innominate artery) was end-to-side anastomosed to the ascending graft in ideal site (Fig. 2c). Antegrade bilateral cerebral and upper extremity perfusion was not interrupted in these three stages. On completion of this final anastomosis, active rewarming continued until the patient could be weaned from CPB.

### Variables and statistical analysis

Patients were followed-up by cardiologists. Telephone interview of patients was performed. The follow-up period was 30 days after operation. We defined operative mortality as death within 30 days of operation. Neurological dysfunction was defined as any new clinically or radiographically evident brain injury present after the operation, especially the temporary or permanent paresis or paralysis that resolved before hospital discharge. Acute renal failure was defined as the need to initiate renal support with hemofiltration. Statistical analysis was performed using statistical software SPSS 16.0 for Windows 7. When the quantitative variables obeyed normal distribution, they were presented as mean ± SD and analyzed by the t-test. Skewed quantitative variables were presented as median and interquartile range, and analyzed by the rank-sum test. Univariate analysis of operative mortality, acute renal failure, neurological dysfunction and respiratory support was performed by the X^2^ test.

## Results

### The modified Y-graft technique using FAB has advantages in shortening the systemic circulatory arrest time and aortic clamp time, and its short-term outcomes are comparable or superior to the traditional Y-graft technique

Twenty-three acute type A aortic dissection patients underwent modified Y-graft technique using FAB. The operative and postoperative data indicating short-term outcomes, including the CPB time, systemic circulatory arrest time, aortic clamp time, cerebral circulatory arrest time, the skin-to-skin time, etc., were collected (Table 2).

**Table 2:** Traditional Y-Graft techniques vs Modified Y-Graft techniques utilizing Femoral artery bypass (FAB)

Variates	Traditional Y-Graft techniques	Modified Y-Graft techniques utilizing Femoral artery bypass (*n* = 23)
Operative variables		
Perfusion and ischemic times, minutes		
Cardiopulmonary bypass	141 (118–183) [1]	236 (200–290)
	236.2 ± 52.5 [7]	
	239 ± 53.1 [8]	
	279 ± 82 [9]	
	273 ± 79 [10]	
Systemic circulatory arrest	65 (51–84) [1]	20.6 ± 6.9
	31.2 ± 6.6 [7]	
	78 ± 34 [9]	
	69 ± 22 [10]	
Aortic clamp	53.9 ± 41.1 [1]	105 ± 25.7
	144 ± 55 [9]	
	163 ± 54 [10]	
Cerebral circulatory arrest	0	0
Lowest nasopharyngeal/rectal temperature, °C	22.0 °C (19.1–23.7) [1]	28 °C
	12.8 ± 2.2 °C (9.9–19.8) [7]	
	15.8 ± 2.1 °C (12.0–22.1) [8]	
	24.0 ± 2.2 °C [9]	
	16–20 °C [11]	
	23 °C [10]	
Concomitant aortic valve replacement	40% [1]	(*n* = 8)34.8%
	2.8% [7]	
Concomitant coronary artery bypass	9% [1]	(*n* = 3)13.0%
	2.8% [7]	
Outcomes		
Operative mortality	2% [1]	(*n* = 2)8.7%
	4.6% [7]	
	4.7% [8]	
	4.9% [9]	
	6.8% [12]	
	2% [13]	
	6.8% [11]	
	6.8% [10]	
Neurologic dysfunction	5% [1]	(*n* = 3)13.0%
	9.4% [8]	
	9.8% [9]	
	14% [14]	
	5% [13]	
	9.1% [11]	
Respiratory support	>2 days 20–51% [10]	>2 days (*n* = 15) 65.2%
	>5 days 5–30% [10]	>5 days (*n* = 8)34.8%
Acute renal failure	11% [1]	(*n* = 5)21.7%
	3.7% [7]	
	6% [8]	
	13.1% [9]	
	5.4% [11]	

Data are reported as mean ± SD, median (interquartile range), or number (%). The superscript numbers are the sequence number of references

Among the 23 patients who underwent FAB technique, two died within 30-days (8.7%), due to pulmonary infection. Two patients were paralyzed from the waist down, one of them was transient, while the other did not recover during the follow-up period. Hemodialysis was performed for five patients (21.7%) during the follow-up period. Fifteen patients (65.2%) received respiratory support for more than 2-days and eight patients (34.8%) for more than 5-days. As for the traditional Y-graft technique, we reviewed previous articles and obtained the corresponding data (shown in Table 2). The results showed that the follow-up data in the patients using FAB modified Y-graft technique were comparable or superior to the data in patients with traditional Y-graft technique. The systemic circulatory arrest time and aortic clamp time were significantly shorter in the FAB modified group.

When using FAB in this surgery, the right brachiocephalic and left common carotid arteries can be reconstructed without CPB and cross-clamping the aorta, so the systemic circulatory arrest time and aortic clamp time are considerably shortened. Besides, all these procedures were performed at room temperature. Compared with the traditional Y-graft technique, the supra-aortic branches can be safely reconstructed in an unhurried fashion, especially if the surgeon is inexperienced. It was easy to repair a leak immediately after anastomoses of the supra-aortic arteries because of optimal mobility and slight wall tension of the anastomoses.

### Perioperative variables and short-term outcomes are superior in modified Y-graft technique using FAB and OSCA

Perioperative demographics of the patients, such as age (46.1 ± 8.3 years), male gender (78.6%), weight (80.8 ± 12.6 Kg), Marfan syndrome (7.1%), aortic valve regurgitation (28.6%), smoking, past or current (75.0%), hypertension (92.9%), renal dysfunction (7.1%), and pulmonary disease, are shown in Table 3.

**Table 3:** One minute systemic circulation arrest (OSCA)

Variates	Modified Y-Graft techniques utilizing Femoral artery bypass (without OSCA, *n* = 23)	Modified Y-Graft techniques utilizing Femoral artery bypass (with OSCA, *n* = 28)	*P* value
Preoperative characteristics			
Age, years	46.5 ± 11.8	46.1 ± 8.3	*P* > 0.05
Male sex	(*n* = 20)87.0%	(*n* = 22)78.6%	*P* > 0.05
Weight, Kg	80.5 ± 13.4	80.8 ± 12.6	*P* > 0.05
Marfan syndrome	(*n* = 2)8.7%	(*n* = 2)7.1%	*P* > 0.05
Aortic valve regurgitation	(*n* = 10)43.5%	(*n* = 8)28.6%	*P* > 0.05
Diabetes mellitus	(*n* = 1)4.3%	(*n* = 0)0%	*P* > 0.05
Smoking, past or current	(*n* = 20)87.0%	(*n* = 21)75.0%	*P* > 0.05
Hypertension	(*n* = 22)95.7%	(*n* = 26)92.9%	*P* > 0.05
Renal dysfunction	(*n* = 1)4.3%	(*n* = 2)7.1%	*P* > 0.05
Pulmonary disease	(*n* = 1)4.3%	(*n* = 0)0%	*P* > 0.05
Operative variables			
Perfusion and ischemic times, minutes			
Cardiopulmonary bypass	236 (200–290)	204 (169–246)	*P* < 0.05
Systemic circulatory arrest	20.6 ± 6.9	1.6 ± 1.1	*P* < 0.05
Aortic clamp	105 ± 25.7	92.9 ± 35.2	*P* > 0.05
Cerebral circulatory arrest	0	0	NA
Skin-to-skin time	486 ± 51.3	432 ± 40.5	*P* > 0.05
Lowest nasopharyngeal temperature, °C	28	32	NA
Concomitant aortic valve replacement	(*n* = 8)34.8%	(*n* = 3)10.7%	*P* < 0.05
Concomitant coronary artery bypass	(*n* = 3)13.0%	(*n* = 1)3.6%	*P* > 0.05
Outcomes			
Operative mortality	(*n* = 2)8.7%	(*n* = 2)7.1%	*P* > 0.05
neurologic dysfunction	(*n* = 3)13.0%	(*n* = 1)3.6%	*P* > 0.05
Respiratory support	>2 days (*n* = 15)65.2%	>2 days (*n* = 10)35.7%	*P* < 0.05
	>5 days (*n* = 8)34.8%	>5 days (*n* = 3) 10.7%	*P* < 0.05
Acute renal failure	(*n* = 5) 21.7%	(*n* = 1)3.6%	*P* < 0.05

*NA* not available. Data are reported as mean ± SD, median (interquartile range), or number (%)

The operative variables are also shown in Table 3, including the median CPB time (204 min), the mean systemic circulatory time (1.6 min), the aortic clamp time (92.9 min), the cerebral circulatory time (0 min), the skin-to-skin time (432 min), and the lowest nasopharyngeal temperature (32 °C). Concomitant procedures included aortic valve replacement (10.7%) and coronary artery bypass grafting (3.6%).

The operative mortality was 7.1%, and neurological dysfunction was 3.6%. Among the 28 patients, 10 patients (35.7%) had >2 days intubation and three patients (10.7%) had >5 days intubation. Only one patient (3.6%) had postoperative renal dysfunction and required temporary hemodialysis before discharge.

### Comparison between the modified Y-graft technique using FAB and modified Y-graft technique using FAB and OSCA

**Fig. 3:**
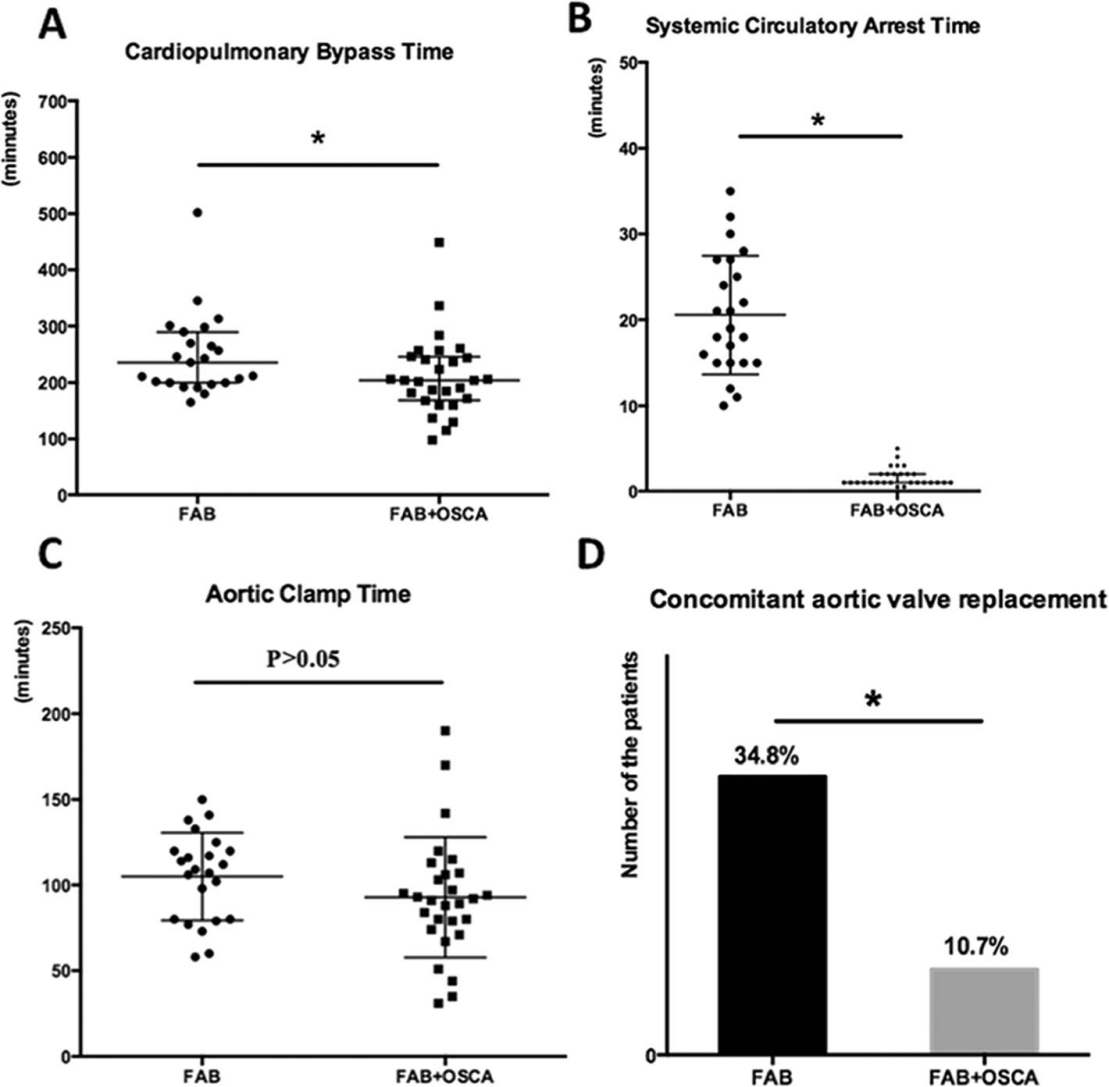
Comparison of the operative variables between the modified Y-graft technique using FAB and modified Y-graft technique using FAB and OSCA. **a**, **b** The cardiopulmonary bypass time [236 (200–290) min, 204 (169–246) min, *p < 0.05] and systemic circulatory arrest time (20.6 ± 6.9 min, 1.6 ± 1.1 min, *p < 0.05) were significantly shortened after modified Y-graft technique using FAB+OSCA technique. **c** The aortic clamp time had no significant difference between the two groups (105 ± 25.7 min, 92.9 ± 35.2 min, *p* > 0.05). **d** The percentage of concomitant aortic valve replacement was lower in the patients with FAB+OSCA techniques (34.8, 10.7%, *p* < 0.05). The error bars in A-C represent mean ± Std. deviation. *p < 0.05, t-test

**Fig. 4:**
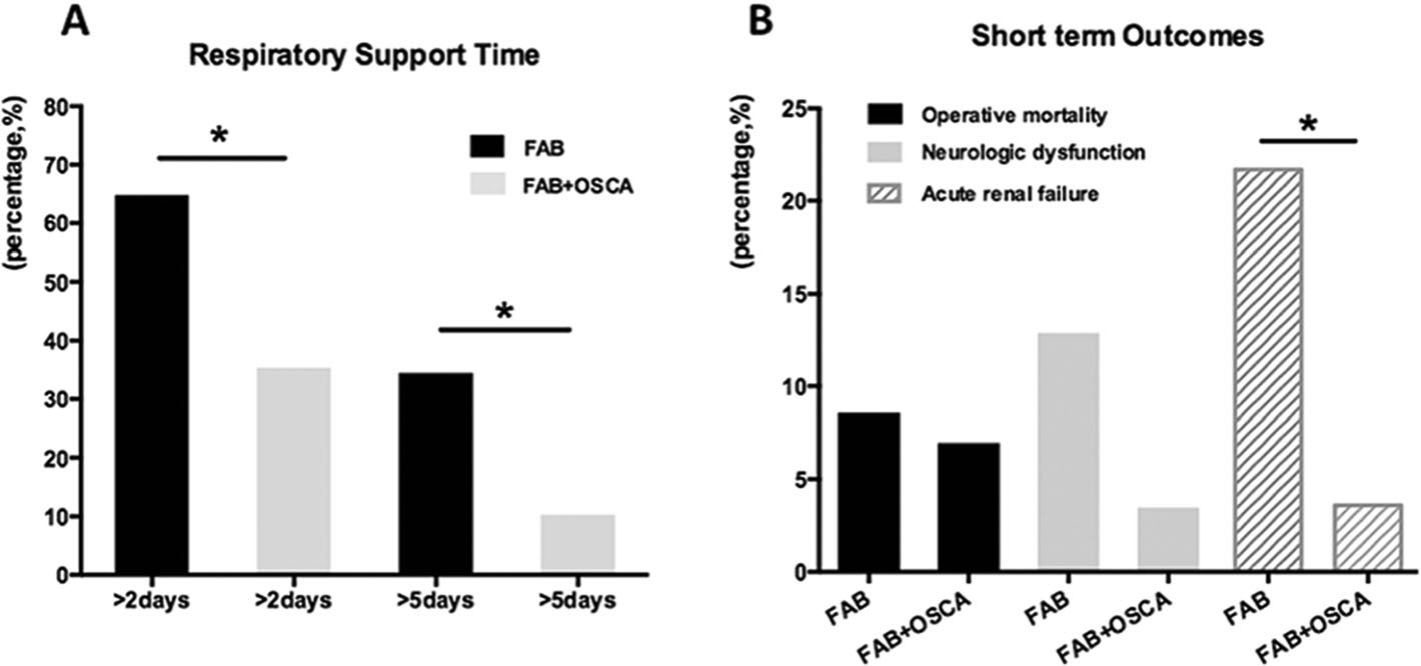
Comparison of the outcomes between the modified Y-graft technique using FAB and modified Y-graft technique using FAB and OSCA. The 28 patients who underwent modified Y-graft technique using FAB+OSCA had an operative mortality of 7.1% and the morbidity of neurological dysfunction was 3.6% (**b**), and these data were statistically equal to the patients who underwent only FAB. Furthermore, the FAB+OSCA patients had significantly shorter respiratory support duration (**a**) and lower morbidity due to acute renal failure (**b**). *p < 0.05, chi-square test

Comparison of these two groups is shown in Table 3. There were no significant differences in preoperative patient characteristics. Compared with the 23 patients who underwent modified Y-graft technique using FAB procedures, the 28 patients who underwent modified Y-graft technique using FAB+OSCA had shorter CPB time (Fig. 3a), systemic circulatory arrest time (Fig. 3b), aortic clamp time (Fig. 3c), and higher lowest nasopharyngeal temperature (32 °C). The percentage of the concomitant aortic valve replacement was also lower in the patients using FAB+OSCA techniques (Fig. 3d). All these differences were statistically significant (*p*<0.05), except for the aortic clamp time and the skin-to-skin time. As for the short-term outcomes, the 28 patients who underwent modified Y-graft technique using FAB+OSCA had an operative mortality of 7.1% and the morbidity of neurological dysfunction was 3.6% (Fig. 4b). These data were statistically equal to the patients who underwent FAB alone. Furthermore, the FAB+OSCA patients had significantly shorter respiratory support duration (Fig. 4a) and lower morbidity due to acute renal failure (*p*<0.05, Fig. 4b).

## Discussion

Aortic arch replacement is one of the most technically challenging cardiovascular operations, incurring considerable risk for perioperative death, stroke, acute renal failure, etc. With the development of the aortic arch replacement surgery, Sun’s procedure has become very popular in China. However, we reviewed numerous articles on Y-graft technique and learnt the operation at the Hartford Cardiovascular Center for 1 year. The Y-graft technique, in which a double Y-graft is used to connect brachiocephalic branches to the main aortic graft, was developed to accomplish arch reconstruction, reduce embolization, and minimize related cerebral ischemia. Recently, we treated 51 cases of acute type A aortic dissection with aortic arch replacement. We modified the procedure of the Y-graft technique and reduced the systemic circulatory arrest time to about a minute.

Originally, we modified the Y-graft technique using the femoral artery bypass (FAB). By this technique, we can reconstruct the brachiocephalic branches at room temperature, without cardiopulmonary bypass (CPB). Even the pericardium does not need to be opened. The left subclavian artery is difficult to expose and anastomose. Nerve injury, especially recurrent laryngeal nerve, can easily occur. Notably, reconstruction of brachiocephalic branches is easier to perform by our modified technique. Besides, it is easy to repair a leak immediately after anastomoses of the supra-aortic arteries because of optimal mobility and slight wall tension of the anastomoses. Twenty-three patients underwent this modified technique. Subsequently, we modified the Y-graft technique using FAB and One Minute Systemic Circulatory Arrest (OSCA) technique. The latter technique majorly decreases the systemic circulatory arrest time, thus reducing the ischemic time of spinal cord and kidneys. Moreover, the lowest nasopharyngeal/rectal temperature can be maintained at 32 °C. To summarize, our modified technique almost eliminated the systemic circulatory arrest time, and reduced the CPB time and other operative variables.

Comparison of the traditional Y-graft technique with the modified Y-graft technique using FAB (Table 2) showed that some of the indicators were not significantly advanced, but our FAB technique makes the operation easier than before. The reconstruction of the supra-aortic arteries can be accomplished in an unhurried fashion and the quality of anastomosis can be improved. It also provides a good surgical visual field for distal aortic anastomosis and eases the blood leak sealing.

Comparison of the FAB technique and FAB+OSCA technique (Table 3) showed that the FAB+OSCA group had significantly shorter CPB time and systemic circulatory time, and significantly higher lowest nasopharyngeal temperature (32 °C). Nevertheless, both operative mortality and morbidity of neurological dysfunction had no statistical differences between the two groups, which may be due to the small sample size. Further research with larger sample size is needed to establish the advantages of FAB+OSCA as a valuable alternative surgical technique for acute type A aortic dissection patients.

## Conclusion

This study clarified the feasibility of FAB+OSCA technique in modifying Y-graft technique. The acute type A aortic dissection patients showed less surgical complications and favorable short-term outcomes by this surgery. Comparison of the traditional Y-graft technique with the modified Y-graft technique using FAB (Table 2) showed that some indicators were not significantly advanced, but our FAB technique makes the operation easier than before. The reconstruction of the supra-aortic arteries can be accomplished in an unhurried fashion, and the quality of anastomosis can be improved. It also provides a good surgical visual field for distal aortic anastomosis and eases the blood leak sealing.

## Data Availability

All data are fully available without restriction.

## Abbreviations

FAB: Femoral Artery Bypass
OSCA: One Minute Systemic Circulatory Arrest
ACT: Activated Clotting Time
